# Lens siderosis resulting from a small intralenticular metallic foreign body

**DOI:** 10.3205/oc000034

**Published:** 2015-11-02

**Authors:** Mehul A. Shah, Shreya M. Shah, Pritesh Teori, Anjli Israni

**Affiliations:** 1 Drashti Netralaya, Dahod, Gujarat, India

## Abstract

We report a rare case of lens siderosis with an undetectable intraocular foreign body by imaging. An 8-year-old boy presented with diminution of vision in the left eye since 3 months. His parents gave a preceding uncertain history of a foreign body injury to his left eye 3 months ago while playing. Presenting visual acuity in the left eye was perception of hand movements. Slit-lamp examination revealed a total white cataract with brownish-pigmented spots on the anterior capsule of the lens, but no intraocular foreign body was found. There was also no evidence of an intraocular foreign body on ultrasonography. Patient underwent cataract extraction with intraocular lens implantation. During the operation, a small (2×1×1 mm in size) intralenticular foreign body of metal material was found and removed carefully with a magnet. The patient regained 20/30 vision after surgery.

## Introduction

Siderosis bulbi is a pigmentary and degenerative change in the eye due to intra-ocular retention of a foreign body containing iron [1]. Clinical findings include iris heterochromia, pupillary mydriasis, iron deposition on corneal endothelium and anterior lens capsule, cataract formation and retinal pigmentary changes [1], [2]. Patients will present with a history of ocular trauma. However, some may be asymptomatic and present later when there is a drop in visual acuity [3].

## Case report

We report a rare case of lens siderosis in a young boy with a preceding history of trauma but no signs of a retained intraocular foreign body.

**Figure 1:**
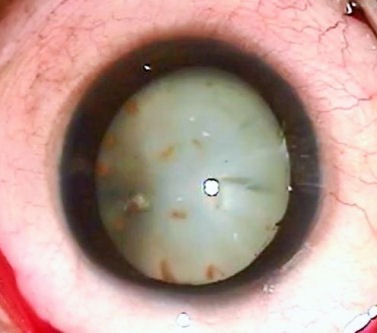
Anterior segment photo of the left eye showing brownish orange deposits on the lens and a white cataract

**Figure 2:**
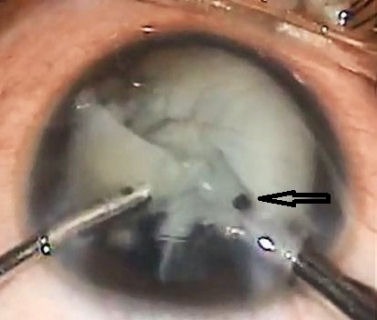
Intralenticular metallic foreign body removed with hand held magnet

An 8-year-old boy presented with diminution of vision in the left eye since 3 months. Presenting visual acuity was hand movement in the left eye. In the left eye, there was a siderotic white cataract and brownish orange pigment clumps on the anterior lens capsule (Figure 1 (Fig. 1)). The anterior chamber was deep and quiet. No intraocular foreign body was visualized via slit-lamp examination. Ultrasound B-scan showed dense cataract with a normal posterior segment with no retinal or vitreous abnormalities. Foreign body was not visible on ultrasonography. The patient underwent a left cataract aspiration and implantation of posterior chamber intraocular lens under general anesthesia. Intra-operatively a small (2×1×1 mm in size) intralenticular metal foreign body was found and removed carefully using a hand held magnet (Figure 2 (Fig. 2)).

**Figure 3:**
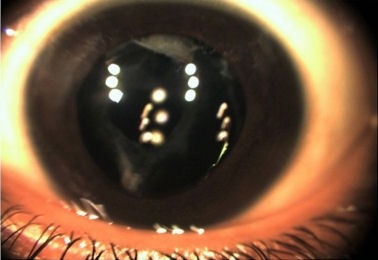
Post operative anterior segment photo of the left eye

Fundus examination was performed post-operatively and there wasn’t any retained intraocular foreign body, retinal detachment, retinal tear, or vitreous hemorrhage. There were no siderotic changes in the retina. The patient regained 20/30 vision after surgery (Figure 3 (Fig. 3)).

## Discussion

Iron is a most common component of metallic intraocular foreign bodies and may lead to ocular siderosis, which presents as reduced visual acuity [1]. Cataract formation may be an indicator of early siderosis and is associated with intralenticular foreign bodies [2]. A retained metallic intralenticular foreign body that is undetectable by imaging modalities is not uncommon [3]. An iron intraocular foreign body can cause siderosis with involvement of the anterior and posterior segment [4]. Management of an intralenticular foreign body may result in good visual outcome [5]. Only a few studies have reported a retained iron intralenticular foreign body undetected by imaging [6]. A retained iron intralenticular foreign body may remain silent for a longer period of time, even up to 60 years [7].

The Current case is rare as the intralenticular foreign body has only affected the crystalline lens in process of siderosis leaving the posterior segment normal.

## Conclusion

This case illustrates the importance of close monitoring of patients with a history of trauma or a previous penetrating injury to the eye, albeit no intraocular foreign body was detected or found, as they might develop ocular siderosis at a later stage. In view of sight-threatening complications of siderosis, prompt intervention is indicated to preserve visual acuity and prevent a progression of siderosis to the posterior segment.

## Notes

### Competing interests

The authors declare that they have no competing interests.
